# Complete mitochondrial genome of *Gortyna guizhouensis* Wu, Yang, and Han, 2022 (Lepidoptera, Noctuidae) from China

**DOI:** 10.1080/23802359.2023.2176183

**Published:** 2023-02-13

**Authors:** Meng Leng, Qiong Liu, Hai-yan Zhao, Xiao-fei Yu, Mao-fa Yang

**Affiliations:** a College of Tobacco Science, Guizhou University, Guiyang, China; b Guizhou Provincial Key Laboratory of Tobacco Quality Research, Guiyang, China; c Guizhou Tobacco Company, Bijie Branch Company, Bijie, China; d Institute of Entomology, Guizhou University, Guiyang, China

**Keywords:** *Gortyna guizhouensis*, tobacco, mitochondrial genome, phylogeny

## Abstract

*Gortyna guizhouensis* Wu, Yang, and Han, 2022 (Lepidoptera, Noctuidae) is a newly discovered moth species that feeds on tobacco piths during the larval stage. In this study, we sequenced and analyzed the complete mitochondrial genome (mitogenome) of *G. guizhouensis* larvae *via* next-generation sequencing. The mitogenome was 15,441 bp long with an overall A + T content of 79.1%, 13 protein-coding genes (PCGs), 22 transfer RNA genes (tRNAs), two ribosomal RNA subunit genes and one control region. The phylogenetic tree, based on the nucleic acid sequences of 13 shared PCGs of 20 Noctuidae species, revealed that *G. guizhouensis* is in a well-supported clade with *Striacosta albicosta*.

## Introduction

**Figure 1.**
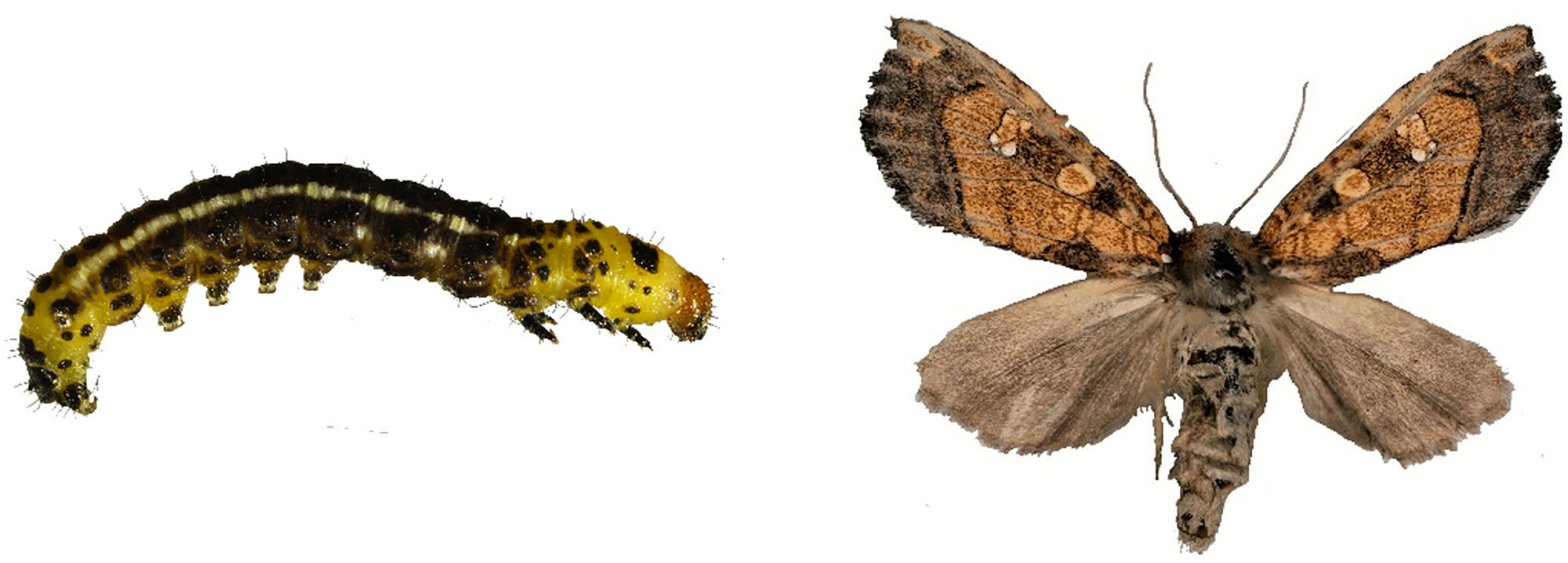
*Gortyna guizhouensis* Wu, Yang, and Han, 2022 (Lepidoptera, Noctuidae): (A) Larva. The larva is black and orange-yellow; (B) Adult. The ground color of the forewings is golden-red and veins are indistinct; a dark streak is present between the orbicular and reniform spots. Photos by Meng Leng.

Tobacco is an important cash crop that, until recently, was host to only one stem borer pest species, *Scrobipalpa heliopa* Loew 1900, in China. However, in 2020, a new tobacco stem borer pest was discovered in Guizhou province. It has been identified as a new species of the Gortyna genus in the Noctuidae family (Noctuoidea superfamily), and has been named *Gortyna guizhouensis* Wu, Yang, & Han, 2022 (Lepidoptera, Noctuidae) (Wu et al. 2022) (Figure 1). The Gortyna genus is widely distributed throughout South Asia, the Middle East, Europe, and North Africa (Gardiner et al. 2017). *G. guizhouensis* is a new species of which no genomic sequencese were available so far, thus mitochondrial genomic data will be conducive to DNA classification and identification of such pests in the future.

## Materials

In this study, we sequenced the complete mitochondrial genome of *G. guizhouensis*. The larvae were collected in Weining County (104°7′57′′E, 27°15′45′′N), Guizhou Province, China in June 2021. Specimens were deposited at the Institute of Entomology, Guizhou University, Guiyang, China (Hai-yan Zhao, haitianyiyan7611@163.com) under the voucher number Gzh-2021-017.

## Methods

We used the DNeasy Blood and Tissue Kit (cat.nos.69504 and 69506, QIAGEN, Hilden, Germany) to extract total DNA from the larvae head. An Illumina ReSeq library was prepared, with an average insert size of 400 bp. The library was sequenced paired-end sequenced (2 × 150 bp) using the Illumina NovaSeq6000 platform (Berry Genomics, Beijing, China). We assembled and annotated the complete mitogenome sequence using NOVOPlasty v2.7.2 (Dierckxsens et al. 2017), with default settings for the K-mer value, and MitoZ v2.4-alpha (Meng et al. 2019) was used to annotate the genome. Samtools v1.1 (Li et al. 2009) was used to assess sequencing depth based on BAM files, and circlize v0.4.16 was used to plot circles.

Phylogenetic analysis was performed with multiple alignments of protein-coding sequences, from the mitochondrial genomes, using PhyloSuite v1.2.2 (Zhang et al. 2020). A phylogenetic tree was constructed using Bayesian inference (BI) in MrBayes (Huelsenbeck and Ronquist 2001). The iTOL webtool (https://itol.embl.de) (Letunic and Bork 2007) was used to display the phylogenetic tree.

## Results

**Figure 2.**
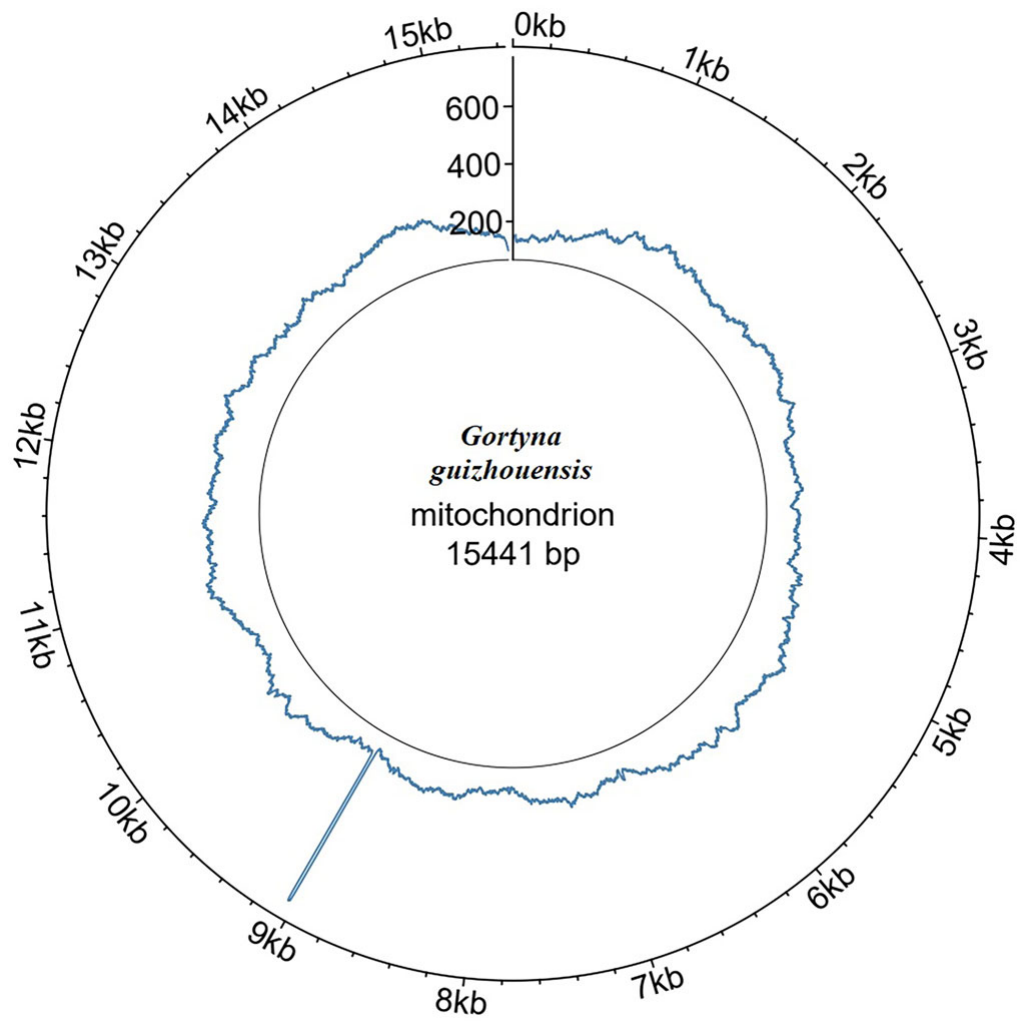
Read coverage map across the *G. guizhouensis* mitochondrial genome. Circlize v0.4.16 was used for plotting.

**Figure 3.**
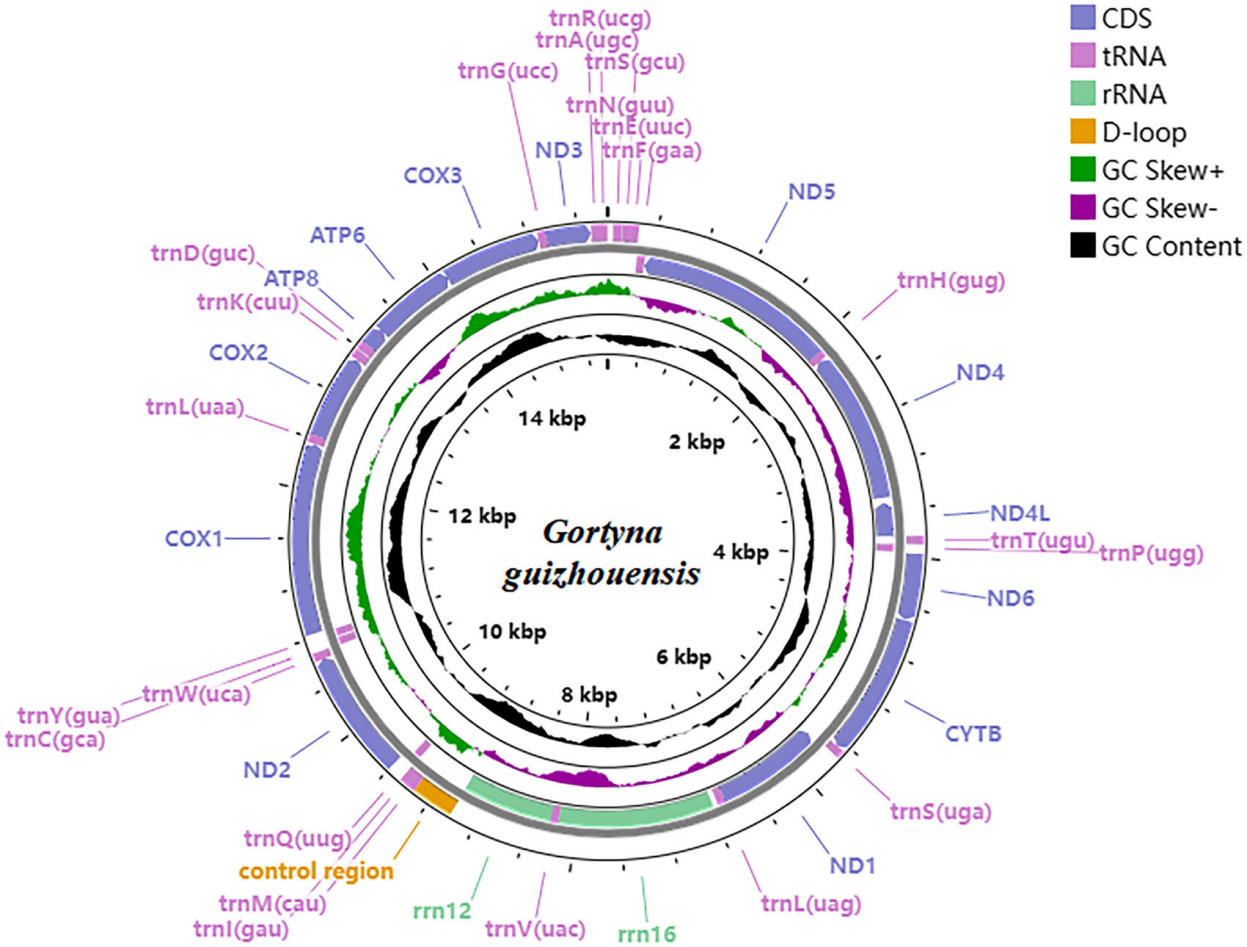
Location and arrangement of genes on the mitogenome of the *G. guizhouensis.* A circular mitochondrial genome map was drawn using the Proksee server (https://proksee.ca/).

The coverage of the *G. guizhouensis* mitochondrial genome was 100%, and the reads in depth were evenly distributed across the mitochondrial genome (Figure 2). The mitochondrial genome of *G. guizhouensis* was 15441 bp long (GenBank accession no. OM140690) and consisted of 13 protein-coding genes (PCGs), 22 transfer RNA (tRNA) genes, two ribosomal RNA (rRNA) genes and one control region (Figure 3). The *16S RNA* and *12S RNA* genes were 1,359 bp and 785 bp long, respectively. The lengths of the 22 tRNA genes ranged from 64 bp (tRNA-Arg) to 72 bp (tRNA-Asp). The overall base composition of the mitogenome was estimated as follows: A 39.7%, T 39.5.4%, C 12.9% and G 8.0%, with a high A + T content of 79.1%. All PCGs started with the standard ATN codons apart from *cox1,* which started with CGA, and *nd1,* which started with TTG, Most of the PCGs terminated with the stop codon TAA, whereas *cox2* and *nd4* terminated with an incomplete stop codon T-.

**Figure 4.**
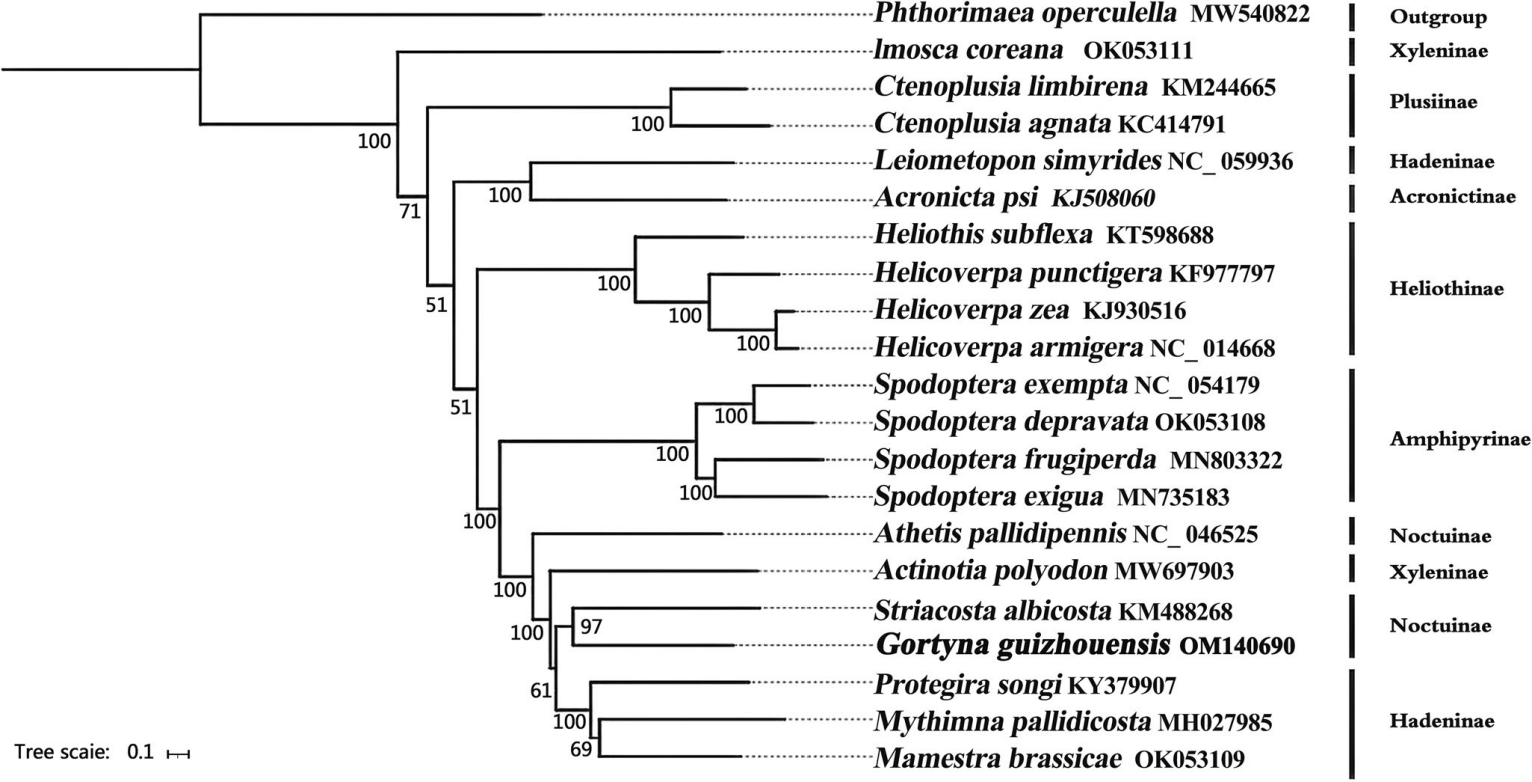
Phylogenetic tree based on 13 mitochondrial protein-coding genes of 21 Lepidoptera species reconstructed by Bayesian inference (BI) method. Numbers on branches are Bootstrap support values (BS). The following sequences were used: MW540822 (Song et al. 2021), OK053111 (unpublished), KM244665 (unpublished), KC414791 (Gong et al. 2013), NC_059936 (unpublished), KJ508060 (unpublished), KT598688 (De Souza et al. 2016), KF977797 (Walsh 2016), KJ930516 (Perera et al. 2016), NC_014688 (Kômoto et al. 2011), NC_054179 (Li et al. 2021), OK053108 (unpublished), MN803322 (unpublished), MN735183 (unpublished), NC_046525 (Li et al. 2020), MW697903 (unpublished), KM488268 (Coates & Abel 2016), KY379907 (Zhao et al. 2022), MH027985 (unpublished), OK053109 (unpublished).

To gain insights into the volution of *G. guizhouensis*, we performed phylogenetic analysis using the complete mitogenomes of 13 PCGs from 21 Lepidoptera species. Of these species, 20 belonged to Noctuidae, and *Phthorimaea operculella* (Gelechiidae: Gelechiinae) was selected as the outgroup. Our analysis revealed that *G. guizhouensis* was closely related to *Striacosta albicosta* within the Noctuidae family. However, the monophyly of Hadeninae, Noctuinae, and Xyleninae could not be confirmed using the phylogenetic tree (Figure 4).

## Discussion and conclusion

The mitochondrial genome of *G. guizhouensis* was 15,441 bp long and comprised 13 PCGs, 22 tRNA genes, two rRNA genes and one control region. In summary, the mitogenome of *G. guizhouensis* can provide the necessary molecular data for further phylogenetic and evolutionary analysis of Noctuidae. This study provides a mitochondrial genomic data for a new tobacco pest, that will be conducive to DNA classification and identification of pests in the future.

## Data Availability

The genome sequence data that support the findings of this study are openly available in GenBank of NCBI at https://www.ncbi.nih.gov under the accession no. OM140690. The associated BioProject, SRA and BioSample numbers are PRJNA826860, SRS12616393, and SAMN27585214, respectively.
